# Relationship between nocturnal blood pressure and 24-h urinary sodium excretion in a rural population in Korea

**DOI:** 10.1186/2056-5909-1-3

**Published:** 2014-09-25

**Authors:** Jinho Shin, Enshi Xu, Young Hyo Lim, Bo Youl Choi, Bae Keun Kim, Yong Gu Lee, Mi Kyung Kim, Mari Mori, Yukio Yamori

**Affiliations:** Department of Internal Medicine, Hanyang University College of Medicine, Seoul, South Korea; Department of Preventive Medicine, Hanyang University College of Medicine, Seoul, South Korea; Division of Cardiology, Department of Internal Medicine, Sung-Ae General Hospital, Seoul, South Korea; Institute for World Health Development, Mukogawa Women’s University, Hyogo, Japan; Division of Cardiology, Department of Internal Medicine, Hanyang University Hospital, Hanyang University College of Medicine, 222 Wangsimni-ro, Seongdong-gu, Seoul 133-791 South Korea

**Keywords:** Sodium, Blood pressure, Urine specimen collection, Blood pressure monitoring, Ambulatory, Rural population

## Abstract

**Background:**

The relationship between sodium intake and blood pressure (BP) is affected by many factors such as absolute level of sodium intake, salt sensitivity, and the accuracy or the timing of the BP measurement. There is no epidemiologic study using both ambulatory BP monitoring (ABPM) and 24-h urine sample in a middle-aged general population.

**Methods:**

In the rural area, Yeojoo County, Gyunggi Province in South Korea, 218 subjects with age between 30 and 59 years old were measured with ABPM and 24-h urine sample. ABPM device was TM2430, and the 24-h urine sample was collected using the aliquot cup. Metabolic syndrome (MetS) score was calculated by the sum of the number of abnormal criteria other than BP.

**Results:**

For both ABPM and 24-h urine sample, 148 subject data was acceptable for the analysis by the creatinine equation and/or the completeness of collection. Age was 47.4 ± 8.3 years (range 30 to 59 years), and female was 85 (57.4%). In multiple linear regression analysis, sodium intake was not an independent factor for casual BPs and daytime BPs whereas sodium intake was an independent factor for nighttime systolic BP (*β* = 1.625, *p* = 0.0026) and nighttime diastolic BP (*β* = 1.066, *p* = 0.0017). When compared to the lowest quartiles of sodium intake, daytime diastolic BP and nighttime BPs were in the higher three quartile groups.

**Conclusions:**

Sodium intake was associated not with casual BPs and daytime BPs but with increased nighttime BPs in the middle-aged general population in Korea.

**Electronic supplementary material:**

The online version of this article (doi:10.1186/2056-5909-1-3) contains supplementary material, which is available to authorized users.

## Introduction

In theory, a bout of increased sodium intake under a given salt excretion capacity would keep the blood pressure (BP) elevated in proportion to the amount of sodium intake until the pressure natriuresis ends. But in population studies, the relationships between the sodium intake and the BP level are weak or mixed unlike the relationship between other factors such as age or obesity and BP level [1, 2]. Even if the reduction of sodium intake is known to be effective in reducing BP regardless of the presence of correlation between sodium intake and BP, the evidence is not sufficient that lower sodium intake improves cardiovascular mortality or outcomes [3, 4]. So clear understanding of the factors related to the relationship between sodium intake and the BP level would make the rationale of the salt reduction strategy in relation to the BP reduction stronger and make it possible to underscore the intervention more specific to a population for whom it would be more beneficial.

Salt resistance or the variation in the salt sensitivity is regarded as the most important explanation for mixed results in the correlation between sodium intake and BP [5]. Salt sensitivity is affected by numerous factors including genetic background (African-American, Asian), age, and obesity [6, 7]. These factors are important factors in demonstrating the relationship between the sodium intake and BP because the measurement of salt sensitivity itself is neither feasible nor reliable in the population study. Metabolic syndrome (MetS) is more specifically known to be involved in salt sensitivity [8].

In addition, there are measurement issues for both the sodium intake and BP. Questionnaire for the sodium intake is often used in the population survey, but, for the Asian diet, it is difficult to ensure the reliability [9]. The main reason is that Asian meal is composed of several recipes including pickled foods and salty soup which are very difficult to quantitate the exact amount of sodium intake by a questionnaire [9].

As for the measurement of BP, the variability of the casual BP makes it harder to show the correlation compared to ambulatory BP monitoring (ABPM) or self-measured BPs. In addition, the BP during daytime and nighttime might have different implications with the sodium intake or neurohumoral axes [10]. Recently, MetS or central obesity is regarded as one of the main factors for increased activity of renin-angiotensin-aldosterone system and/or salt sensitivity [11, 12].

To our best knowledge, there are few studies to investigate the relationship between sodium intake and BP by ABPM and 24-h urine sodium in relatively young- or middle-aged general population [13]. In this study, in order to investigate the relationship between sodium intake and BP in Korean as an Asian population, we examined sodium intake by 24-h urine collection and BP by measuring ABPM for the middle-aged subjects in a rural Korean population.

## Methods

### Study population

By reviewing the study protocol, the two study sites, i.e., Jeomdong-myeon and Ganam-myeon were recommended by the officers in the health-care center in Yeojoo County, Gyunggi province in South Korea, with a rationale that those sites have a relatively high density of the young-aged population and the preserved rural lifestyle. Then, the study protocol was presented in the community leader meeting in the study sites on 13 May 2012. Among 63 communities, 13 communities applied for the study. Most of the reason for the failure to participate was the scarcity of the younger subjects. The population aged between 30 and 59 was 2,710 in the study sites. Among them, 1,179 (43.5%) was estimated as the real habitant by the commercial statistics. Because most of those young people were commuting for their job (68.1%), only 374 subjects were estimated to have a rural lifestyle in the study site.

The participant was recruited by audible public announcement in the community offices. Two hundred and thirteen subjects, i.e., 149 subjects from Jeomdong-myeon and 64 subjects from Ganam-myeon, were recruited during June 2012 to January 2013. Because of the diversity of the habitant status and the commuting for the job, it was limited to recruit the subjects with the eligible age who actually live in the study area during daytime for their living. On the base of daily living and the activity in the community, the community leaders estimated that at least 50% of the community members of the age group participated for the study.

### Study protocol

**Figure 1:**
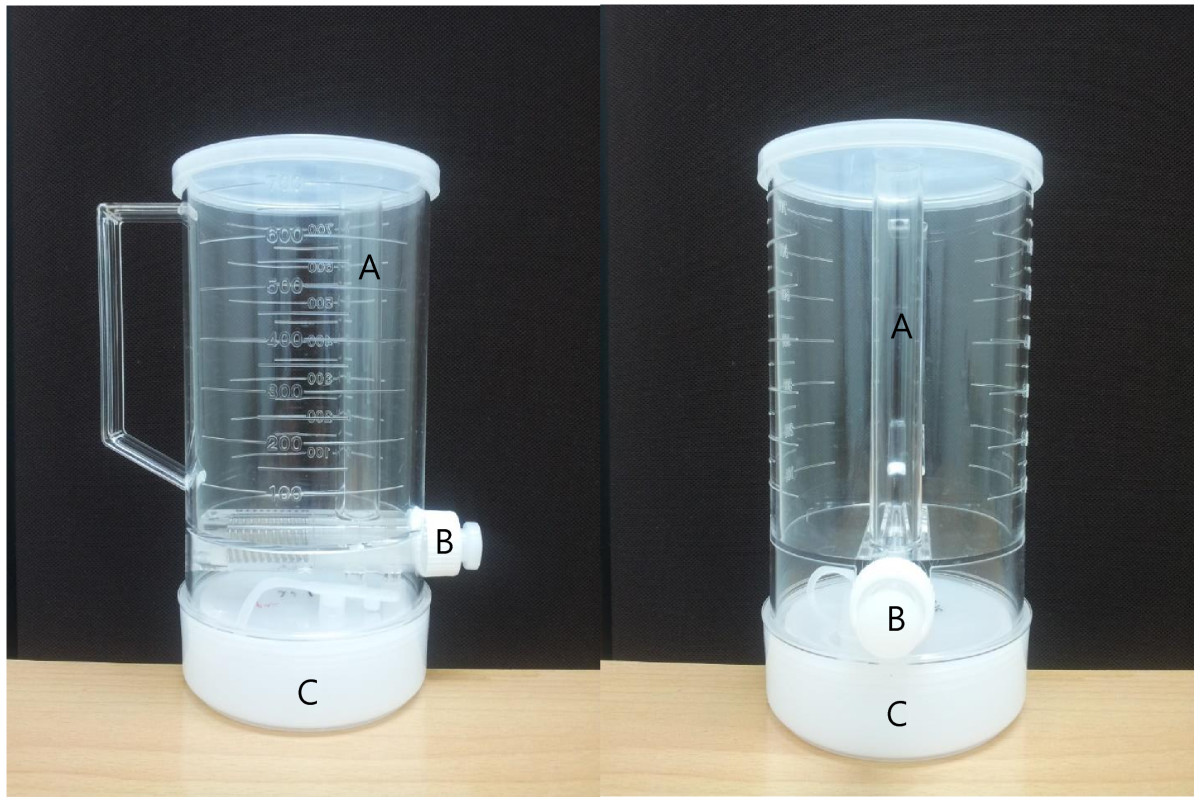
**Aliquot cup to collect 1/40 of each voided urine. (A)** Hollow tube to separate 1/40 of the voided urine in the cup. **(B)** The button to send down the separated urine into the separated container. **(C)** Smaller and sealed container to collect all of the 1/40 urine during study period.

The study was performed at the two community health-care centers affiliated to the two myeon districts. On the first day of the survey, after getting informed consent, casual BP was measured and ambulatory BP monitoring device was applied. The subject was educated and tested for the use of the aliquot cup to collect 24-h urine. The aliquot cup was designed to collect 1/40 of each voided urine by pressing the collecting button (Figure 1) [14]. In this study, sodium intake was defined as the amount of sodium excretion in 24-h urine sample. Tests for urine sample were performed in the World Health Research Institute in Mukogawa Women’s University by sending a frozen urine sample via FedEx postal service. Tests for blood sample were performed both in the domestic central laboratory (SCL, Seoul, Korea) and central laboratory in Japan for standardization for the 30 subjects during the first session. After the standardization, all the blood tests were performed in the domestic laboratory. Collaborating researcher (YY) joined domestic researchers during the survey for initial 30 subjects to standardize the study protocol.

### Health examination and blood chemistry

Abdominal circumference was measured using spring-loaded tape ruler (SECA, Hamburg, Germany) according to the standardized manner in which the ruler was placed horizontally at the mid-level between lower margin of the ribs and the iliac crest at the end of normal expiration in a standing position. BP was measured two times with 1 min interval after 5 min rest in sitting position using HEM 907 (Omron, Kyoto, Japan). The average value was used for systolic blood pressure (SBP) and diastolic blood pressure (DBP). Blood samples were collected in the morning of the first day of the survey after 12-h fasting to measure fasting blood glucose, lipid profiles, creatinine, and hemoglobin A1C.

When taking antihypertensive medication by questionnaire or when SBP or DBP is 140/90 mmHg or higher, hypertension was diagnosed. Diabetes mellitus was diagnosed when fasting blood glucose was 126 mg/dL, higher or hemoglobin A1C was 6.5% or higher or taking antidiabetic medication. According to the definition of MetS of the International Diabetes Federation in 2005, those with MetS must show central obesity, which is defined by cutoff values of 90 and 85 cm for waist circumference in men and women, respectively [15]. Irrespective of whether patients was taking lipid-lowering drugs and/or oral hypoglycemic agents or not, MetS was defined in individuals with central obesity plus two or more of the following: elevated triglyceride level (≥1.7 mmol/L, 150 mg/dL), reduced high-density lipoprotein cholesterol (<1.0 mmol/L [40 mg/dL] in men and <1.3 mmol/L [50 mg/dL] in women), elevated arterial pressure of at least 130/85 mmHg or antihypertensive medication, elevated fasting plasma glucose (≥5.6 mmol/L, ≥100 mg/dL), and previously diagnosed type 2 diabetes [16]. MetS score was defined as the summed number of abnormal criteria for MetS except for the BP criteria to avoid double counting in addition to the other parameters of BP.

### Collection of the 24-h urine sample

There were technical failures for the use of aliquot cup in 25 subjects, and 7 subjects refused to collect 24-h urine sample. Among 181 subjects completing the 24-h urine, 111 samples were judged as a valid sample by applying the creatinine equation. The creatinine amount should be within the range between 14.4 and 33.6 mg/kg in male and between 10.8 and 25.2 mg/kg in female [17]. Because the equation was not drawn from the study population or Korean population, for the rest of the sample, the completeness of the urine collection was assessed by additional interview for urine collection. Additional 51 samples which were collected completely with an exception for the skipped collection during defecation were considered as valid samples.

### Ambulatory blood pressure monitoring

TM2430 (A&D, Tokyo, Japan) was used for ABPM. The measurement intervals were 30 min during both daytime and nighttime. There were 10 subjects who refused to get ABPM. Non-compliances during measurement were noted in 9 subjects mainly due to the inability to do shower, poor sleep, and unexpected schedule. There were 11 device failures or insufficient number of valid readings less than 14 for the day and 7 for the night. [18] The criteria for error readings were 1) SBP less than 70 mmHg or higher 250 mmHg, 2) DBP less than 40 mmHg or higher than 150 mmHg, and 3) pulse pressure less than 20 mmHg or higher than 150 mmHg [19]. Finally, 148 subjects with valid data for ABPM data and 24-h urine collection were analyzed.

BPs for daytime and nighttime were defined using narrow fixed interval methods in which daytime and nighttime BPs were averaged based on the readings between 8 AM and 9 PM and midnight and 5 AM, respectively. The dipper was defined as the decrease in SBP by 10% or more during the nighttime compared to daytime in the narrow fixed time interval method. The study was in compliance with the Helsinki Declaration, and the protocol was approved by the Institutional Review Board in Hanyang University Seoul Hospital (HYUH 2012-05-021-001).

### Statistical analyses

The sample size was 63 for each group to detect the 5 mmHg difference in the mean BP with standard deviation of 10 mmHg between high vs. low sodium intake groups to expect 0.80 as study power and 0.05 as type I error. For descriptive analysis, paired *t*-test was performed to compare casual BP and ambulatory BP and otherwise, Student’s *t*-tests for difference between the genders. To analyze the role of sodium intake on the level of BP, general linear model was used for casual BP, daytime and nighttime BP. Independent variable was sodium intake measured by 24-h urine study. Covariates such as age, sex, and MetS score were included in the model. For comparison among quartile groups defined by sodium intake, general linear model was used for least squares means adjusted for the covariates and linear trend was tested using linear contrast function with or without stratification by MetS. Multiple comparisons between groups were adjusted by Tukey’s correction. Multiple logistic regression analysis for non-dipper was performed to calculate odds ratio of the independent variables such as age (per 10 years), female, sodium intake (per gram), MetS, hypertension, diabetes mellitus, and smoking. Data was expressed as mean ± standard deviation and least squares means ± standard error for unadjusted and adjusted means, respectively. *p* value less than 0.05 was considered as statistically significant. *R* was used for the graphic, and SAS ver. 9.3 (SAS Institute Inc., Cary, NC, USA) was used for the statistical analysis.

## Results

### General characteristics

The age of the population was 47.4 ± 8.3 years, and the older subjects were higher than those younger subjects (chi-square, *p* < 0.0001). As shown in Table 1, casual SBP and DBP were higher in the male subjects. Daytime SBP and DBP were higher casual SBP and DBP (*p* < 0.0001 and *p* < 0.0001, respectively). Fasting blood glucose was higher in the male subjects. Triglyceride was also higher in the male subjects, but the prevalence of MetS (*p* = 0.26) or MetS score (*p* = 0.83) was not different between genders. Sodium intake measured by 24-h urine sample was 4.9 ± 2.4 g in men and 3.8 ± 2.4 g in women (*p* = 0.0075). Sodium to potassium ratio in moles was higher in men than those in women. Nighttime BP was lower in women, so the nocturnal dipping and the prevalence of dipper were higher in women than in men.

**Table 1:** **General characteristics of the study population according to gender**

Variable	Male (*n* = 63)	Female (*n* = 85)	*p*value
Age (year)	48.3 ± 8.5	47.4 ± 8.2	0.53
30–39	10	14	0.71
40–49	20	32	
50–59	33	39	
Casual SBP (mmHg)	127.9 ± 14.5	119.0 ± 16.4	0.0008
Casual DBP (mmHg)	77.2 ± 11.8	71.3 ± 11.3	0.0002
Pulse rate (beats per minutes)	68.2 ± 11.5	67.2 ± 10.2	0.59
Hypertension (%)	25 (39.6)	24 (28.2)	0.143
Waist circumference (cm)	88.2 ± 7.0	86.4 ± 7.9	0.141
Body mass index (kg/m^2^)	25.6 ± 3.2	24.7 ± 3.2	0.103
Fasting blood glucose (mg/dL)	98.1 ± 16.4	89.8 ± 13.9	0.0012
Diabetes mellitus (%)	6 (9.5)	4 (4.7)	0.24
Current smoking (%)	22 (34.9)	1 (1.2)	<0.0001
Total cholesterol (mg/dL)	183.2 ± 40.0	189.3 ± 34.0	0.29
Triglyceride (mg/dL)	158.9 ± 96.7	119.4 ± 71.9	0.0044
High density lipoprotein (mg/dL)	47.5 ± 10.3	56.4 ± 12.9	<0.0001
Creatinine (mg/dL)	1.1 ± 0.1	0.9 ± 0.1	<0.0001
MetS (%)	17 (26.9)	19 (22.3)	0.51
MetS score	1.49	1.25	0.23
0	15	27	0.62
1	20	27	
2	14	15	
3	10	14	
4	4	2	
24-h urine sample			
Sodium (g/day)	4.9 ± 2.4	3.8 ± 2.4	0.0075
Potassium (g/day)	2.6 ± 0.9	2.4 ± 1.3	0.275
Sodium/potassium	3.3 ± 1.3	2.8 ± 1.1	0.0051
Ambulatory blood pressure monitoring			
SBP, daytime (mmHg)	131.7 ± 10.8	130.8 ± 15.1	0.681
SBP, nighttime (mmHg)	113.9 ± 15.0	107.9 ± 17.0	0.0291
SBP, 24 h (mmHg)	124.9 ± 11.4	121.2 ± 14.3	0.0938
DBP, daytime (mmHg)	83.0 ± 7.5	81.6 ± 9.5	0.3389
DBP, nighttime (mmHg)	72.7 ± 10.2	66.5 ± 10.1	0.0004
DBP, 24 h (mmHg)	78.9 ± 7.5	75.1 ± 8.5	0.0056
Nocturnal dipping (%)	13.6 ± 7.9	17.3 ± 9.3	0.011
Dipper (%)	46 (73)	69 (81)	0.22

Values are presented as mean ± standard deviation or number (%).

*SBP* systolic blood pressure, *DBP* diastolic blood pressure, *MetS score* the summed number of abnormal criteria for metabolic syndrome except for blood pressure.

### Sodium intake and the level of blood pressure

**Figure 2:**
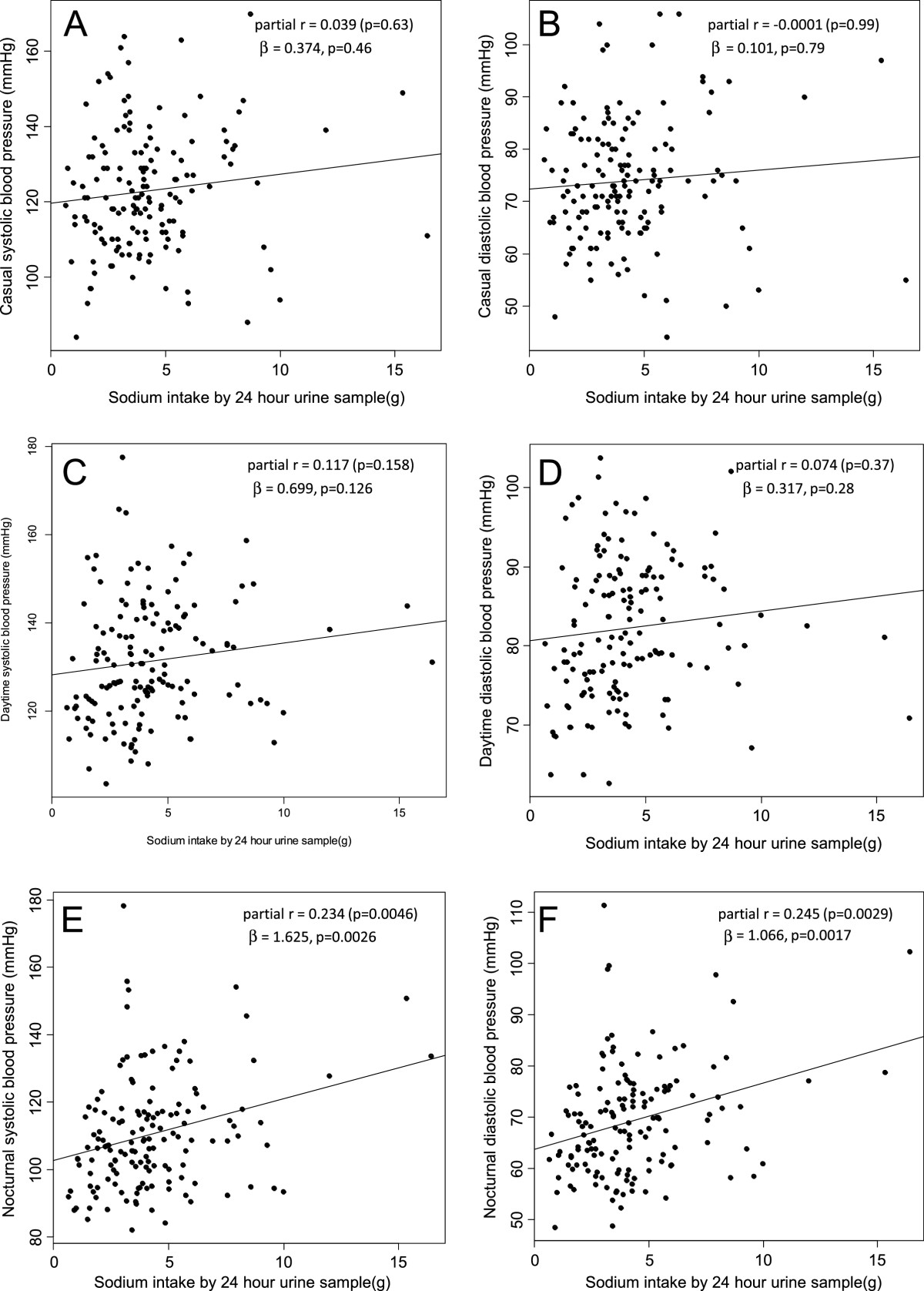
**Plots for sodium intake measured by 24-h urine sample and casual and ambulatory blood pressures.** In panel **(A)** to **(D)**, sodium intake was not independently associated with blood pressures whereas as shown in panel **(E)** and **(F)**, sodium intake was independently associated with nighttime blood pressures. *β* and *p* values were derived from the multiple linear regression model including age, sex, sodium intake, and metabolic syndrome score.

Partial Pearson’s correlation coefficients between sodium intake and BPs adjusted for age, sex, and MetS score were 0.039 for casual SBP (*p* = 0.63), −0.0001 for casual DBP (*p* = 0.998), 0.117 for daytime SBP (*p* = 0.158), 0.074 for daytime DBP (*p* = 0.37), 0.234 for nighttime SBP (*p* = 0.0046), and 0.245 for nighttime DBP (*p* = 0.0029) (Figure 2). In the general linear model as shown in Table 2, MetS score was positively associated with both casual SBP and DBP. And the female gender was negatively associated casual BPs. But sodium intake was not independently associated with casual BP (Table 2). In regards to ambulatory BPs, sodium intake was positively associated only with nighttime BPs in systole and diastole additionally to MetS score (Table 2). In multiple linear regression analysis, as shown in Figure 2, sodium intake was not an independent factor for casual SBP (*β* = 0.374, *p* = −0.46), casual DBP (*β* = 0.101, *p* = 0.79), daytime SBP (*β* = 0.699, *p* = 0.126), and daytime DBP (*β* = 0.317, *p* = 0.28) whereas sodium intake was an independent factor for nighttime SBP (*β* = 1.625, *p* = 0.0026) and nighttime DBP (*β* = 1.066, *p* = 0.0017).

**Table 2:** **Parameter estimates in general linear model showing the factors contributing to casual and ambulatory BPs in mmHg**

Variable	Casual BP (mmHg)		Ambulatory BP (mmHg)			
	SBP	DBP	Daytime BP		Nighttime BP	
			SBP	DBP	SBP	DBP
Sodium intake						
Age	−0.036	−0.131	0.038	0.003	0.072	0.002
Sex	−7.880*	−5.321*	0.157	−0.720	−3.539	−4.660*
Sodium (g)	0.303	−0.041	0.673	0.287	1.573*	1.036*
MetS score	3.313*	2.771*	1.317	1.421*	2.604*	1.474*
*R*sq	0.134	0.145	0.031	0.051	0.127	0.169
Sodium potassium ratio						
Age	−0.048	−0.163	0.044	0.031	0.107	0.023
Sex	−8.434*	−5.915*	−0.518	−1.017	−4.757	−5.516*
Na/K	−0.416	−0.994	0.081	0.018	0.844	0.459
MetS score	3.398*	2.889*	1.391	1.454*	2.702*	1.549*
*R*sq	0.134	0.153	0.017	0.045	0.077	0.115

*BP* blood pressure, *SBP* systolic blood pressure, *DBP* diastolic blood pressure, *MetS score* the summed number of abnormal criteria for metabolic syndrome except for BP, *Rsq R*-squared, *Na*/*K* sodium potassium ratio.

*p < 0.05.

### Comparison among groups by the amount of sodium intake

When the subject was divided by the median value of the sodium intake (3.8 g) as higher and lower groups, there was no significant difference in adjusted BPs for age, sex, and MetS score, i.e., casual SBP (123.0 ± 1.8 vs. 126.3 ± 2.0, *p* = 0.23), casual DBP (75.5 ± 1.5 vs. 78.0 ± 1.6, *p* = 0.19), daytime SBP (134.6 ± 1.8 vs. 131.0 ± 2.0, *p* = 0.13), daytime DBP (84.7 ± 1.1 vs. 82.3 ± 1.3, *p* = 0.13), nighttime SBP (113.1 ± 2.1 vs. 111.2 ± 2.4, *p* = 0.49), and nighttime DBP (70.8 ± 1.3 vs. 69.9 ± 1.5, *p* = 0.62).

**Figure 3:**
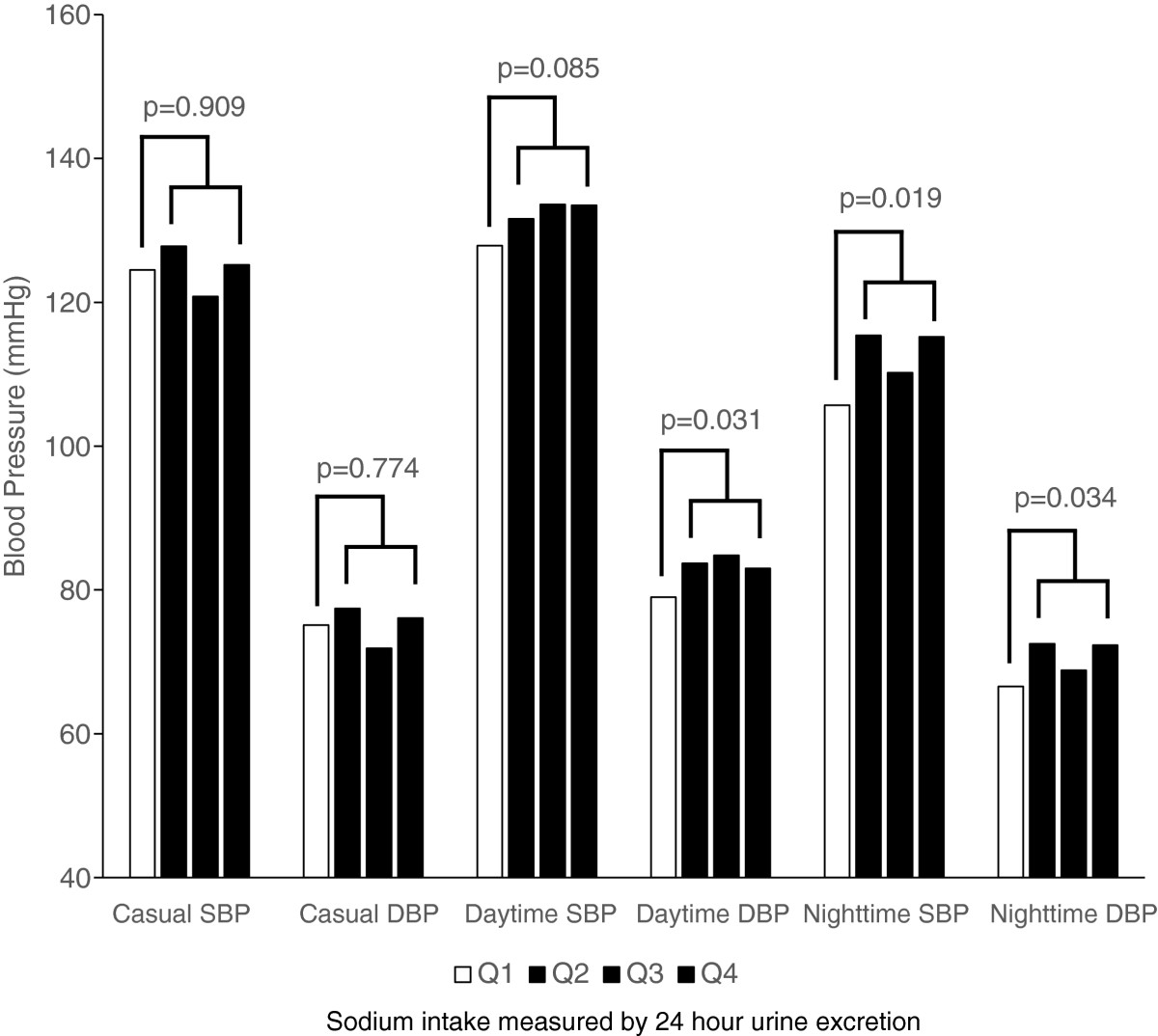
**Comparison between the lowest quartile group (Q1) for sodium intake and the rest of the quartile groups (Q2–Q4).** The significant differences were observed in daytime diastolic blood pressure and nighttime blood pressures. *p* values adjusted for age, sex, and metabolic syndrome score.

When the subject was divided according to the quartiles of sodium intake as shown in Table 3, both the differences among groups and the linear trends of increasing BP as the increased sodium intake was noted in daytime DBP, nighttime SBP, and night DBP only when they were unadjusted or adjusted for age and sex. When further adjusted for MetS score, the linear trends were borderline in statistical significance. When comparing the first quartile group with the rest of the subject, age, sex, and MetS score adjusted daytime DBP, nighttime SBP, and nighttime DBP were significantly higher in the group (Q2, Q3, and Q4; *n* = 108) taking sodium more than 2.65 g compared to Q1 (Figure 3).

**Table 3:** **Comparison of BP levels among the quartile groups by sodium intake measured by 24-h urine sample in general linear models**

Variable	Sodium excretion quartiles				*p*for analysis of variance	*p*for trend
	Q1 (<2.65 g)	Q2 (2.65–3.8 g)	Q3 (3.8–5.25 g)	Q4 (>5.25 g)		
Unadjusted						
Casual SBP	119.7 ± 2.7	125.1 ± 2.7	120.4 ± 2.7	126.1 ± 2.7	0.22	0.22
Casual DBP	72.0 ± 1.9	75.4 ± 1.9	71.3 ± 1.9	76.7 ± 1.9	0.13	0.23
Daytime SBP	127.2 ± 2.2	131.3 ± 2.2	132.9 ± 2.2	133.6 ± 2.2	0.17	0.038
Daytime DBP	78.1 ± 1.4	83.3 ± 1.4	84.2 ± 1.4	83.1 ± 1.4	0.011	0.0127
Nighttime SBP	102.9 ± 2.6	113.8 ± 2.6	109.4 ± 2.6	115.8 ± 2.6	0.0034	0.0039
Nighttime DBP	64.2 ± 1.7	71.4 ± 1.7	68.7 ± 1.7	72.8 ± 1.7	0.003	0.0023
Adjusted for age and sex						
Casual SBP	122.6 ± 2.7	126.7 ± 2.6	119.3 ± 2.6	125.4 ± 2.6	0.19	0.95
Casual DBP	73.8 ± 2.0	76.6 ± 1.9	70.6 ± 1.8	76.3 ± 1.9	0.09	0.85
Daytime SBP	127.0 ± 2.3	131.1 ± 2.3	133.0 ± 2.2	133.6 ± 2.2	0.19	0.0413
Daytime DBP	78.2 ± 1.5	83.3 ± 1.4	84.2 ± 1.4	83.1 ± 1.4	0.018	0.019
Nighttime SBP	104.4 ± 2.7	114.6 ± 2.6	108.8 ± 2.6	115.5 ± 2.6	0.01	0.0275
Nighttime DBP	65.9 ± 1.7	72.0 ± 1.7	68.1 ± 1.7	72.4 ± 1.6	0.0148	0.0455
Adjusted for age, sex, and MetS						
Casual SBP	124.5 ± 2.7	127.8 ± 2.6	120.8 ± 2.6	125.2 ± 2.5	0.29	0.68
Casual DBP	75.1 ± 2.0	77.4 ± 1.9	71.9 ± 1.9	76.1 ± 1.8	0.18	0.78
Daytime SBP	127.9 ± 2.4	131.6 ± 2.2	133.6 ± 2.3	133.5 ± 2.2	0.28	0.075
Daytime DBP	79.0 ± 1.5	83.7 ± 1.4	84.8 ± 1.4	83.0 ± 1.4	0.034	0.055
Nighttime SBP	105.7 ± 2.8	115.4 ± 2.6	110.2 ± 2.7	115.2 ± 2.6	0.03	0.065
Nighttime DBP	66.6 ± 1.8	72.5 ± 1.7	68.8 ± 1.7	72.3 ± 1.7	0.036	0.089

*BP* blood pressure, *SBP* systolic blood pressure, *DBP* diastolic blood pressure, *MetS score* the summed number of abnormal criteria for metabolic syndrome except for BP.

## Discussion

The main finding of our study is that sodium intake measured by 24-h urine study is associated only with nighttime BP in a middle-aged general population in a rural area in Korea. This result was paralleled with the findings that the 1 g sodium intake increases the chance of non-dipper by 21.2%. But in these age ranges between 30 and 59, the association between age and the BP regardless of measurement methods was not statistically significant.

These findings suggest that the inconsistent results in the previous study for the relationship between sodium intake and the BP could be attributable to the limitation of the BP measurement by casual BP or daytime BP, measurement of sodium intake, and/or the masking effect of age factor which could be exaggerated in an elderly population. In this study, female gender was associated with casual BPs in contrast to the non-significant association in daytime ambulatory BP, which was higher than the casual BPs. This finding suggests that a masked effect of casual BPs may be factors preventing the relationship between sodium intake and BP from being demonstrated. The finding that higher daytime ambulatory BP than the casual BPs was consistent with the previous study result showing high prevalence of masked hypertension which was performed in another rural population in Korea [20]. Physical activity related to the rural lifestyle might be speculated as possible explanations for this finding. The study protocol to stop physical activity when the ABPM device starts to work may not be sufficient to acquire resting BP.

Even though the present study showed that nighttime BPs are more sensitive in the relationship with sodium intake than casual BPs, further study with a larger sample size is needed because the present study may be underpowered to demonstrate the casual BPs and sodium intake. In another reason, the linear relationship could be demonstrated in a study with a larger sample size. In our study, the linear trends in the quartile group comparison were not statistically significant as shown in Table 3. Such non-linearity can be attributable to the finding that the BPs in the second quartile group were comparable or higher than those in the third quartile group. The non-linearity in the relationship between sodium intake and the BP was previously reported in the international study especially when the covariates were properly adjusted [21]. The cutoff value of sodium intake less than 2.65 g between the first quartile and the rest of the quartiles is similar to the previously reported threshold of 100 mmol of sodium [22]. The non-linear relationship was also further supported by the finding that the comparison between groups divided by the cutoff value of 2.65 g sodium intake showed consistently significant difference in the adjusted BP levels as shown in Figure 3. The differences in the statistical significance between multiple linear regression analysis are shown in Figure 2, and the general linear model shown in Table 3 may be the moderation of the effect of the extreme values in the sodium intake or BP in the robust regression analyses. The daytime DBP was significant only in the group comparison between Q1 and Q2 to Q4.

In our study, the prevalence of MetS was comparable to the previous report and MetS or MetS score was the main factor for the BPs both in casual BPs and BPs measured by ABPM [23]. This finding is consistent with the previous study showing the relationship between MetS and BPs despite non-significant relationship between sodium intake by questionnaire and BPs in the Korean National Health and Nutrition Examination Survey data [9]. In spite of a possibility that obvious elevation of BP may be permitted by MetS because of salt sensitivity, there was no significant interaction between them. But more comprehensive study with larger sample size is needed because the subjects with MetS or hypertension could already be trying a lifestyle modification.

The association of sodium intake with nighttime BP may be important, and there may be some clinical implications. Firstly, the nighttime BP is not available in routine clinical practice, and the BP increase related to high sodium intake may be masked for the clinician. Secondly, this relationship may be exaggerated in much elderly subjects that comprise most of the hypertensive population. Thirdly, the nighttime BP was known to be an independent prognostic factor so that the high sodium intake might be an indicator for cardiovascular prognosis. Fourthly, in some recent studies, 24-h BP control is regarded more and more meaningful in hypertension treatment. High sodium intake may be an obstacle to achieve a 24-h BP control.

With the viewpoint of the pressure natriuresis, sodium remained due to ineffective pressure natriuresis or salt sensitivity may increase the nighttime BP to excrete the sodium for maintenance of sodium balance. In the meantime, the increased nighttime BP itself or the condition related to ineffective pressure natriuresis or salt sensitivity could further damage the cardiovascular system [24–26]. Isolated elevation of the nighttime BP presenting as isolated nocturnal hypertension was reported to be associated with cardiovascular events and total mortality [27, 28].

There are some limitations deserve to be mentioned. Firstly, it is cross-sectional study which could not identify causality relationship between MetS and nighttime BP. Secondly, the representativeness of the subject for the study population might be limited due to the small number considering the registered population in the study area. The study area which was a typical rural area in which the lifestyle had been changing rapidly especially in young people. Because of the job, study candidates worked outside of the city by commuting or by separate living during weekdays from their family. Because the author tried to examine the lifestyle in the rural area, subjects who were away from the area or not available for the study in the morning hours were not considered for the study. Therefore, considering the small number of the subject who was living in their rural lifestyle, the author thought that the present study was not subject to serious selection bias. Thirdly, the sample of 24-h urine was classified as acceptable using both considering excreted creatinine amount and the questionnaire. Such classification was defined because some participants argued that their collection was complete and because there are protocols which using such combined criteria [29]. The technical failure of aliquot cup was present, and it was thought to be due to a cold weather in a single survey day. So the author would regard the 24-h urine study as acceptable despite such limitations. Fourthly, the influence of alcohol intake was not properly adjusted which was reported important as a covariate [21].

## Conclusions

In conclusion, sodium intake measured by 24-h urine was an independent factor for daytime DBP, nighttime SBP, and nighttime DBP. This study is the first study showing the relationship between sodium intake measured by 24-h urine study and the nighttime BP in a middle-aged general population in the rural area. Nighttime blood pressure checked by ABPM might be useful when to identify the elevation or possibly decrease of blood pressure by salt intake.

## Authors’ original submitted files for images

Below are the links to the authors’ original submitted files for images. Authors’ original file for figure 1 Authors’ original file for figure 2 Authors’ original file for figure 3

## Abbreviations

ABPM: ambulatory blood pressure monitoring
BP: blood pressure
DBP: diastolic blood pressure
MetS: metabolic syndrome
SBP: systolic blood pressure.
