# Erratum to: Relationship between nocturnal blood pressure and 24-h urinary sodium excretion in a rural population in Korea

**DOI:** 10.1186/s40885-015-0025-6

**Published:** 2015-06-18

**Authors:** Jinho Shin, Enshi Xu, Young Hyo Lim, Bo Youl Choi, Bae Keun Kim, Yong Gu Lee, Mi Kyung Kim, Mari Mori, Yukio Yamori

**Affiliations:** Department of Internal Medicine, Hanyang University College of Medicine, Seoul, South Korea; Department of Preventive Medicine, Hanyang University College of Medicine, Seoul, South Korea; Department of Internal Medicine, Division of Cardiology, Sung-Ae General Hospital, Seoul, South Korea; Institute for World Health Development, Mukogawa Women’s University, Hyogo, Japan; Department of Internal Medicine, Division of Cardiology, Hanyang University Hospital, Hanyang University College of Medicine, 222 Wangsimni-ro, Seongdong-gu Seoul, 133-791 South Korea

After publication of the article [1] it was discovered that a citation error had occurred, resulting in different citation numbers in the PDF and HTML files. The correct citation of 20:9 has now been updated in all versions of the manuscript. We apologise for any inconvenience caused by this error.
