# Supplementary figures and images for: Relationship between nocturnal blood pressure and 24-h urinary sodium excretion in a rural population in Korea
Source: Clin Hypertens. 2014 Sep 25;20:9. doi: 10.1186/2056-5909-1-3 (PMC4763416; doi:10.1186/2056-5909-1-3)

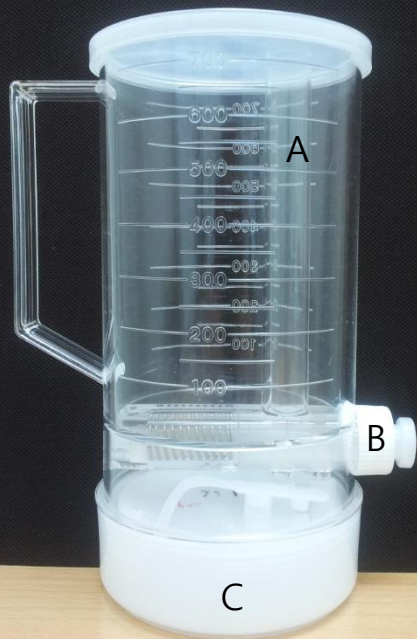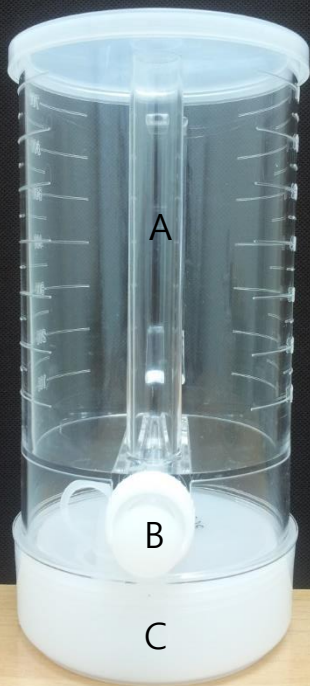

Supplement: Supplementary file 1 — Authors’ original file for figure 1 [file 40885_2014_3_MOESM1_ESM.pdf]

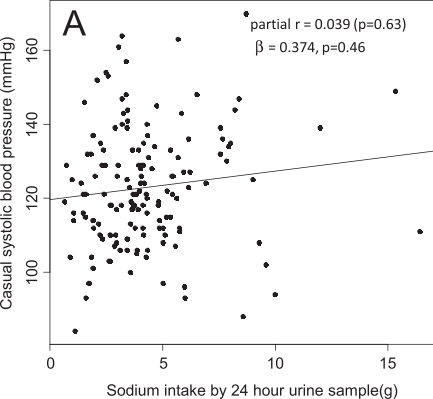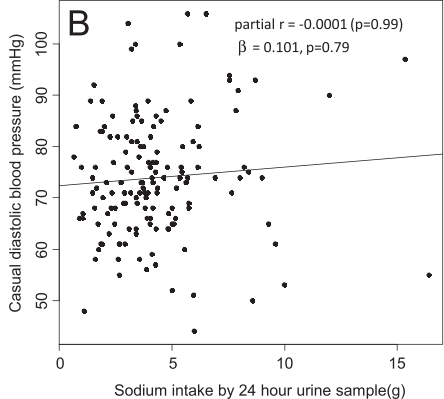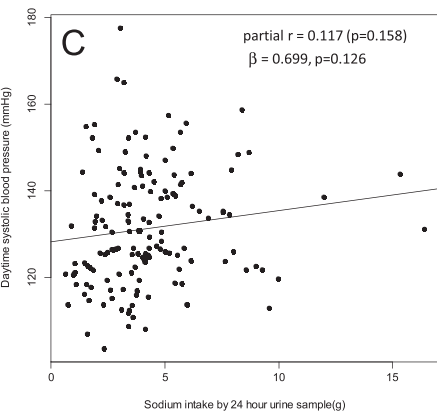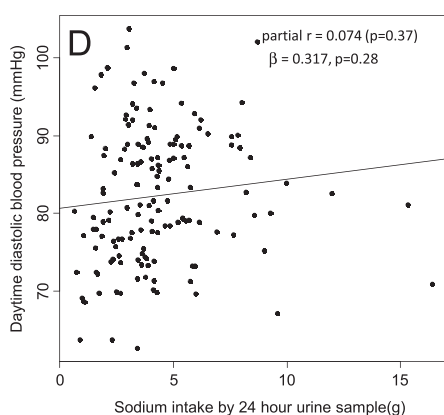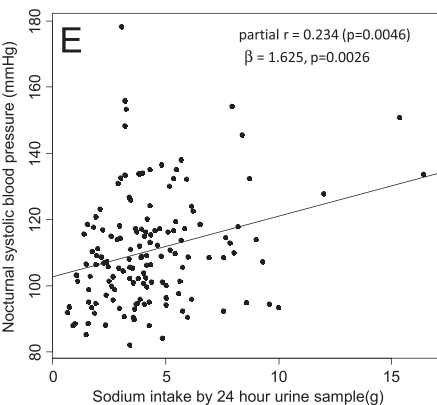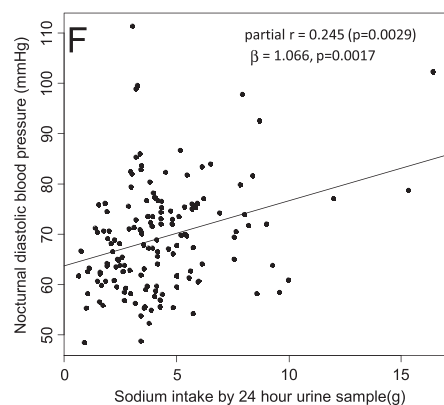

Supplement: Supplementary file 2 — Authors’ original file for figure 2 [file 40885_2014_3_MOESM2_ESM.pdf]

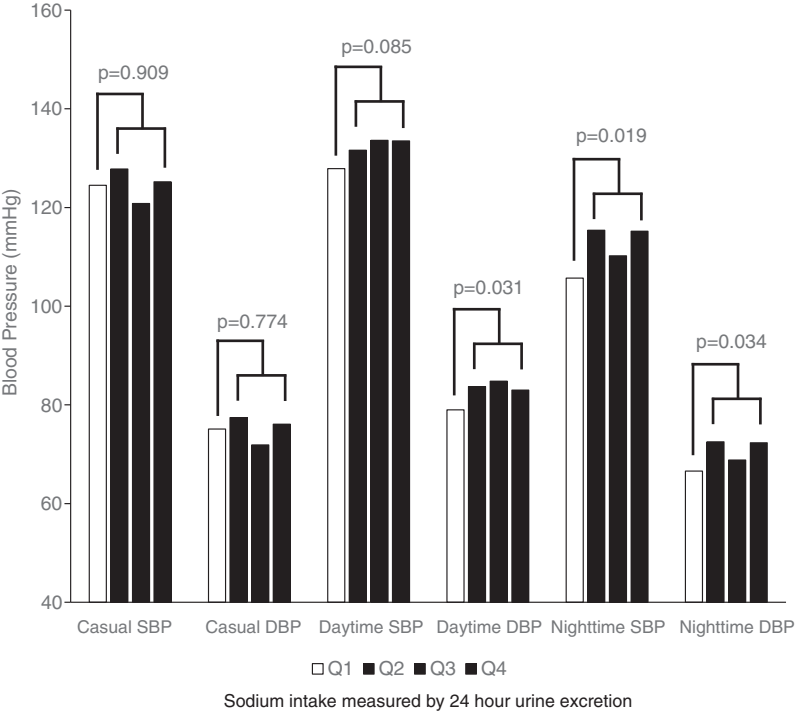

Supplement: Supplementary file 3 — Authors’ original file for figure 3 [file 40885_2014_3_MOESM3_ESM.pdf]
